# Mechanistic Insights Into House Dust Mites Immunotherapy‐Induced Desensitization for Sustained Protection of Allergic Rhinitis in Mice

**DOI:** 10.1155/adpp/7002987

**Published:** 2026-05-24

**Authors:** Ahmad Dzulfikri Nurhan, Yu-Wen Cheng, George Hsiao, Honey Dzikri Marhaeny, Yusuf Alif Pratama, Chrismawan Ardianto, Mahardian Rahmadi, Junaidi Khotib

**Affiliations:** ^1^ School of Pharmacy, College of Pharmacy, Taipei Medical University, Taipei, 11031, Taiwan, tmu.edu.tw; ^2^ Department of Pharmacy Practice, Faculty of Pharmacy, Airlangga University, Surabaya, 60115, Indonesia, unair.ac.id; ^3^ Department of Pharmacology, School of Medicine, College of Medicine, Taipei Medical University, Taipei, Taiwan, tmu.edu.tw; ^4^ Biomaterial Translational Research Group, Faculty of Pharmacy, Airlangga University, Surabaya, 60115, Indonesia, unair.ac.id; ^5^ Biomedical Pharmacy Research Group, Faculty of Pharmacy, Airlangga University, Surabaya, 60115, Indonesia, unair.ac.id

**Keywords:** allergic rhinitis, chronic respiratory disease, desensitization, house dust mite, immunomodulation, mouse model

## Abstract

The worldwide prevalence of allergic rhinitis (AR) is substantial and is considered a global health problem. Allergen immunotherapy (AIT) is the only approach that treats the underlying cause of allergy by inducing a desensitization response to the allergen. In this study, we investigated the effectiveness and the possible desensitization mechanisms of house dust mite (HDM) allergen extract‐based IT against mice models of AR. The experimental mice were divided into six groups: normal group, normal group with HDM challenge, AR group, and three AR groups that received HDM‐IT at low, moderate, and high doses. The effectiveness of HDM‐IT was evaluated based on nose‐rubbing and sneezing behavior, IL‐4 mRNA expression, eosinophil infiltration into nasal mucosal tissue, serum IgE levels, serum IgG2a levels, and serum IgE/IgG2a ratio. This study discovered that the administration of HDM‐IT reduced nose‐rubbing and sneezing behavior, decreased IL‐4 mRNA expression and eosinophil infiltration into nasal mucosal tissue, decreased serum IgE levels, increased serum IgG2a levels, and decreased serum IgE/IgG2a ratio in AR mice. Thus, HDM‐IT effectively promotes a desensitization response to HDM allergens in mouse models of AR.

## 1. Introduction

Allergic rhinitis (AR) is a chronic inflammatory disease of the nasal mucosa and is recognized as part of the broader spectrum of chronic respiratory diseases mediated by hypersensitivity reactions [1, 2]. This disease represents a major global health concern, with a rising prevalence in many countries [3]. Approximately 25% of the worldwide population is affected [4]. Beyond its high prevalence, AR significantly impairs patient’s quality of life, affects their work and social life, causes various comorbidities, and burdens the healthcare system [5, 6].

Environmental factors are central to the etiology of AR [7]. Among them, HDMs are the leading allergens responsible for triggering hypersensitivity reactions in AR [8, 9]. The most dominant HDM species, *Dermatophagoides pteronyssinus*, contains a protein with a high allergenicity known as Der p1 [10, 11].

The allergenic effect of Der p1 is attributed to its potent proteolytic activity, which disrupts epithelial barrier integrity by cleaving tight junction proteins such as occludin and claudins, thereby facilitating allergen penetration and enhancing antigen presentation [11, 12]. Beyond barrier disruption, HDM proteases exert multiple immunopathogenic effects: they cleave surfactant proteins in the airway epithelium, target immune receptors expressed on dendritic, B, and T cells, activate damage‐associated molecular patterns (DAMPs), and protease‐activated receptors (PARs) on epithelial cells [11, 13].

In individuals who experience hypersensitivity, HDM exposure elicits both acute and chronic allergic reactions. Acute‐phase allergic reactions are characterized by symptoms such as itching of the nose, sneezing, rhinorrhea, and nasal congestion [5, 14]. The chronic phase of allergic reactions is characterized by persistent infiltration of proinflammatory cells (e.g., eosinophils, mast cells, and T lymphocytes) and increased production and release of proinflammatory cytokines (e.g., IL‐4, IL‐5, and IL‐13) within the nasal mucosa. These mediators sustain mucosal inflammation, drive tissue damage, and exacerbate disease severity [15, 16].

Current AR management focuses primarily on symptomatic control with antihistamines, leukotriene receptor antagonists, and corticosteroids. However, these drugs do not address the underlying cause of the disease [17, 18]. Allergen immunotherapy (AIT) is the only class of therapy that treats the underlying cause of allergies. AIT acts through defined immunological mechanisms, inducing allergen‐specific tolerance (desensitization) [19, 20].

The efficacy of AIT depends critically on the quality and standardization of allergen extracts [21]. These extracts, derived from relevant biological sources, must accurately reflect the immunogenic properties of the causative allergen. Given the prominent role of HDM in AR pathogenesis, standardizing HDM extracts represents a promising strategy for optimizing AIT [22, 23]. Notably, allergen extracts from different regions or manufacturers often show variable allergenic potency [24, 25]. Such differences may arise from protein polymorphisms, variation in source materials, and inconsistencies in extract composition [26–28].

To address the need for causative therapy to treat allergy‐related diseases, including AR, we examined the potency and desensitization mechanisms of the HDM allergen extract as immunotherapy in a mouse model of AR.

## 2. Materials and Methods

### 2.1. Experimental Animals

A total of 48 female Balb/c mice in healthy condition, aged 6–8 weeks and weighing 20–25 g, exhibiting normal behavior and no visible body abnormalities, were used for this study. The mice were acclimated for 7 days before administering treatment, placed in ventilated cages under a 12‐h light/12‐h dark cycle, and at a temperature of 24°C ± 2°C with access to standard pelleted laboratory food and drinking water *ad libitum*. Maintenance and all treatment of experimental animals were carried out at the Animal Laboratory Research Centre at the Faculty of Pharmacy, Universitas Airlangga, Surabaya, Indonesia.

### 2.2. Reagents

This study used the allergen extract of *Dermatophagoides pteronyssinus* (HDM) 5 mg/mL from the Teaching Industry Allergen, Dr. Soetomo General Academic Hospital, Surabaya, Indonesia [24, 29]. The extract was quality‐controlled for protein content (containing Der p1 with 11.3–26.6 ng/mL of concentration), ensuring batch consistency and standardization [24, 29]. HDM allergen extract and aluminum hydroxide were dissolved in normal saline (0.9% NaCl). The aluminum hydroxide (Merck), which was used as an adjuvant for induction in experimental animals, was mixed with the HDM allergen extract in normal saline.

### 2.3. Animal Grouping and Treatment Protocol

**FIGURE 1:**
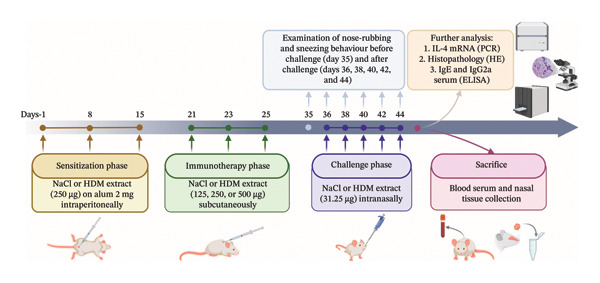
Treatment protocol for experimental animals.

Mice were randomly divided into six treatment groups. The first group was a normal group of mice, the second group was normal mice challenged with HDM allergen extract, and the third group included mice with AR. The fourth to sixth groups included mice with AR that were administered HDM‐IT at low, medium, and high doses. All experimental groups were treated in three phases: sensitization, IT, and challenge. For the sensitization phase, the mice were injected intraperitoneally (i.p.) with normal saline (200 μL) or HDM allergen extract (250 μg) plus aluminum hydroxide (2 mg) in 200 μL of normal saline according to the treatment group on Days 1, 8, and 15. For the IT phase, mice were injected subcutaneously (s.c.) with normal saline (200 μL) or HDM allergen extract at low (125 μg), moderate (250 μg), or high (500 μg) doses in 200 μL normal saline according to the treatment group on days 21, 23, and 25. Finally, in the challenge phase, the mice were administered normal saline or HDM allergen extract (31.25 μg) in 25 μL of normal saline intranasally (i.n.) on days 36, 38, 40, 42, and 44. The treatment protocol is illustrated in Figure 1.

### 2.4. Examination of AR Manifestation

Nose‐rubbing and sneezing behavior were assessed in the mice by recording video for 15 min on the 35th day (as a control without challenge) and on Days 36, 38, 40, 42, and 44 (shortly after the challenge administration). Nose‐rubbing and sneezing frequency was calculated by two observers blinded to the grouping of the animals in this experiment [24, 30].

### 2.5. Measurement of IL‐4 mRNA Expression in the Nasal Mucosal Tissue

Nasal mucosal tissue samples were collected after the sacrifice was performed for each group of mice on the 45th day (24 h after the final i.n. challenge). Total RNA was extracted from the nasal mucosa cells using the Total RNA Purification Kit (Jena Bioscience, Germany), and the concentration of the extracted RNA was measured using the QuantiFluor RNA Sample Kit (Promega, USA). First‐strand cDNA was synthesized using the GoScript TM Reverse Transcription System (Promega, USA) using a thermal cycler (Applied Biosystems, USA). The following were the primers used in this study: IL‐4 (forward: 5′‐TCG​GCA​TTT​TGA​ACG​AGG​TC‐3′; reverse: 5CTG​TGG​TGT​TCT​TCG​TTG​CTG‐3′) and β‐actin (forward: 5′GGC​CAA​CCG​TGA​AAA​GAT​GA‐3′; reverse: 5CAC​GCT​CGG​TCA​GGA​TCT​TC‐3′). RT‐qPCR was done using the GoTaq Master Mix (Promega, USA) and the MyGo Mini tool (IT‐IS Life Science Ltd.). IL‐4 expression was normalized to β‐actin expression and presented as a fold increase relative to the expression of the normal group (control).

### 2.6. Histopathological Evaluation of the Nasal Mucosal Tissue

On the 45th day (24 h after the last intranasal challenge), after the mice were sacrificed, the heads were fixed in 10% neutral‐buffered formalin at room temperature for 7 days. A coronal incision was then made on the nasal mucosal tissue with a thickness of 4 μm, placed on slides, and stained with hematoxylin and eosin. The slides were examined under an optical microscope, and a digital image was captured at 400x magnification. Eosinophil infiltration in the nasal mucosal tissue of the mice was calculated based on the average of five random areas of the nasal mucosal tissue. Observations were made by a histopathologist blinded to the treatment groups in this experiment [31, 32].

### 2.7. Measurement of Serum Immunoglobulin (Ig) Levels

The serum Ig levels for IgE and IgG2a were measured using an ELISA. Blood samples were collected by intracardiac puncture under anesthesia on day 45 (24 h after the final intranasal challenge). The serum was separated from whole blood to measure serum IgE levels using the Mouse IgE ELISA kit (MBS704547, MyBioSource Inc.), and serum IgG2a levels were measured using the Mouse IgG2a ELISA kit (MBS56078, MyBioSource Inc.) based on the manufacturer’s instructions. Data was collected using a microplate reader (Biochrom EZ Read 2000, Cambridge, UK).

### 2.8. Data Analysis

Data were tested for normality using the Shapiro–Wilk test and for homogeneity of variances using Levene’s test. As these assumptions were met, one‐way or two‐way ANOVA was applied as appropriate, followed by Tukey’s post hoc test. The frequency of nose‐rubbing and sneezing was analyzed using a two‐way ANOVA followed by Tukey’s post hoc test. The pattern of IL‐4 mRNA expression in the nasal mucosal tissue of mice, the number of infiltrating eosinophils in the nasal mucosal tissue, serum IgE levels, serum IgG2a levels, and the ratio of IgE/IgG2a were analyzed by one‐way ANOVA followed by Tukey’s post hoc test. All statistical analyses were performed using GraphPad Prism software (Version 9.0).

## 3. Results

### 3.1. HDM‐IT Reduces the Frequency of Nose‐Rubbing and Sneezing Behavior in AR Mice

To evaluate the severity of AR symptoms, nose‐rubbing and sneezing behaviors were analyzed in different experimental groups before and after repeated HDM challenges. Nose‐rubbing behavior (Figure 2(a)) was significantly increased in the AR group compared to the Normal and Normal + HDM Challenge groups at all challenge time points (*p* < 0.01). Notably, IT at all doses significantly reduced nose‐rubbing behavior compared to the AR group (*p* < 0.05 or *p* < 0.01), with the high‐dose IT group exhibiting the most pronounced reduction.

FIGURE 2Nose‐rubbing and sneezing frequencies in allergic rhinitis mice following repeated HDM challenges. (a) Nose‐rubbing and (b) sneezing in experimental animals. ^∗∗^ *p* < 0.01 versus the normal group; ^##^ *p* < 0.01 versus the normal group + HDM challenge; ^Δ^ *p* < 0.05 and ^ΔΔ^ *p* < 0.01 versus the allergic rhinitis group. Each bar shows the mean ± SEM of 8 mice/group. Statistical tests were performed using a two‐way ANOVA. HDM, house dust mite allergen extract; IT, HDM immunotherapy.

**Figure:**
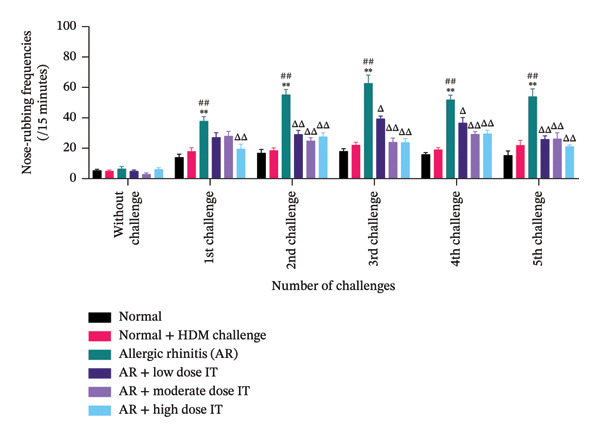
(a)

**Figure:**
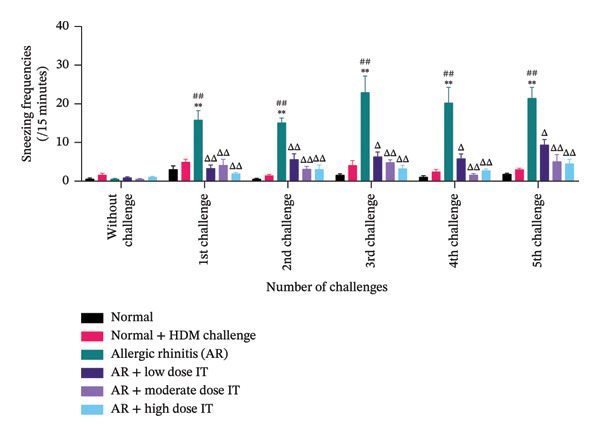
(b)

Similarly, sneezing frequency (Figure 2(b)) was markedly elevated in the AR group following the HDM challenge (*p* < 0.01 vs. Normal and Normal + HDM Challenge groups). IT effectively attenuated sneezing behavior, with moderate and high doses of IT significantly reducing sneezing episodes compared to the AR group (*p* < 0.05 or *p* < 0.01). The high‐dose IT group demonstrated the greatest suppression of sneezing frequency, approaching levels observed in the Normal + HDM Challenge group.

These findings suggest that HDM‐induced AR significantly increases nasal symptoms, and AIT effectively mitigates these allergic responses dose‐dependently (Figure 2).

### 3.2. HDM‐IT Attenuates IL‐4 mRNA Expression in the Nasal Mucosal Tissue

**FIGURE 3:**
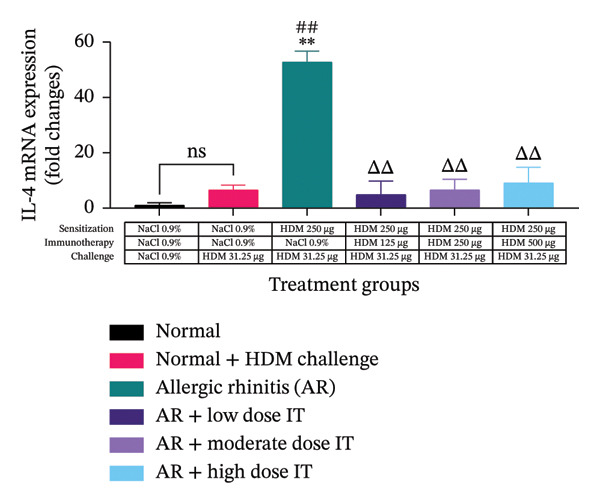
IL‐4 mRNA expression in the nasal mucosal tissue following HDM‐induced AR and IT treatment. ns (nonsignificant) indicates that there was no significant difference; ^∗∗^ *p* < 0.01 versus the normal group; ^##^ *p* < 0.01 versus the normal group + challenge HDM; ^ΔΔ^ *p* < 0.01 versus AR group. Each bar shows the mean ± SEM of 3 mice/group. Statistical tests were performed using a one‐way ANOVA.

To assess the impact of HDM‐induced AR and IT on Th2‐mediated inflammation, IL‐4 mRNA expression levels in nasal tissue were analyzed using quantitative RT‐PCR (Figure 3). IL‐4 mRNA expression was significantly upregulated in the AR group, showing a marked increase compared to the Normal and Normal + HDM Challenge groups (*p* < 0.01). This result confirms that HDM exposure induces a robust Th2‐driven allergic response.

IT significantly suppressed IL‐4 mRNA expression compared to the AR group (*p* < 0.01) across all tested doses. However, IL‐4 expression in the IT‐treated groups remained slightly elevated compared to the Normal group, suggesting partial but effective immune modulation.

These findings highlight that AIT effectively attenuates HDM‐induced IL‐4 expression, demonstrating its potential to shift the immune response away from a Th2‐dominant allergic profile.

### 3.3. HDM‐IT Reduces Eosinophil Infiltration in the Nasal Mucosal Tissue

To determine the role of eosinophils as one of the inflammatory cells in AR and their dynamics after the administration of HDM IT, a histopathological examination of the nasal mucosal tissue of the mice was performed. Based on microscopic observations, the AR mice that were administered medium and high doses of HDM IT, but not low dose, showed a significant decrease in eosinophil cell infiltration compared with the untreated control group of AR mice (Figure 4).

FIGURE 4Eosinophil infiltration in the nasal mucosal tissue following HDM‐induced AR and IT. (a) Representative histopathological features of the nasal mucosal tissue of experimental animals. Slides were stained with hematoxylin and eosin and then observed under a light microscope with a magnification of 400x. Red arrows indicate eosinophil infiltration. (b) A quantitative analysis of eosinophil infiltration in the nasal mucosal tissue of the experimental animals. ns (nonsignificant) indicates that there was no significant difference; ^∗∗^ *p* < 0.01 versus the normal group; ^##^ *p* < 0.01 versus normal group + challenge HDM; ^ΔΔ^ *p* < 0.01 versus AR group. Each bar shows the mean ± SEM of 4 mice/group. Statistical tests were performed using a one‐way ANOVA.

**Figure:**
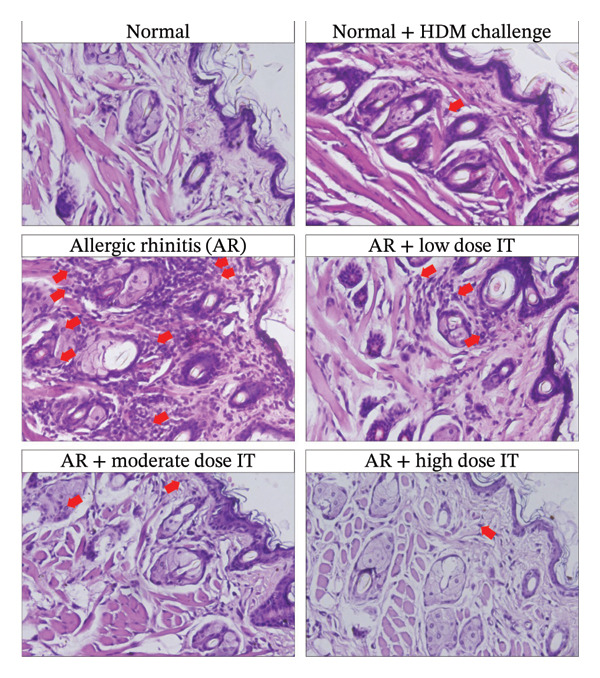
(a)

**Figure:**
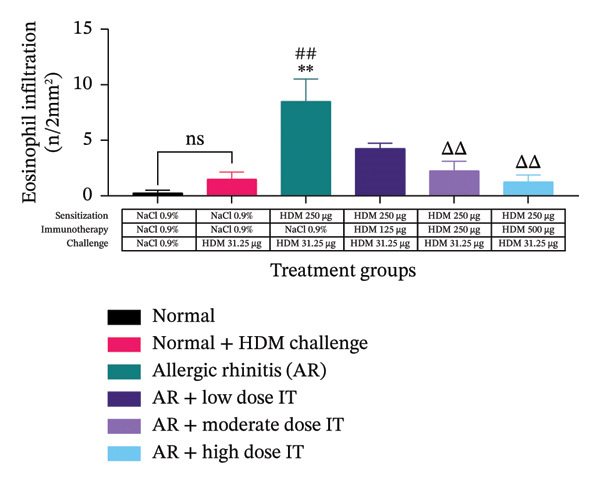
(b)

### 3.4. Effects of HDM‐IT on Serum IgE Levels

The serum IgE and IgG2a and their ratio were assessed to evaluate the humoral immune responses in this study. We observed that serum IgE levels in the mice administered HDM‐IT at low (4.361 ± 0.870 ng/mL), moderate (6.941 ± 1.523 ng/mL), and high (8.511 ± 3.490 ng/mL) doses were significantly lower compared with the control AR mice (37.269 ± 3.232 ng/mL; *p* < 0.01) (Figure 5(a)). Furthermore, we found that the levels of IgG2a in the group of AR mice administered low (10.791 ± 5.977 ng/mL) and moderate (13.101 ± 4.837 ng/mL) doses of IT did not show any significant differences compared with the untreated group of AR mice (15.105 ± 5.836 ng/mL; *p* = 0.999 and *p* > 0.999, respectively). Interestingly, IgG2a levels in the group of AR mice given a high dose of IT (63.111 ± 12.008 ng/mL) exhibited significantly higher IgG2a levels compared with the group of mice with AR (15.105 ± 5.836 ng/mL; *p* < 0.05) (Figure 5(b)).

FIGURE 5Effect of HDM immunotherapy on (a) serum IgE concentration, (b) serum IgG2a concentration, and (c) serum IgE/IgG2a ratio in experimental animals. ns (nonsignificant) indicates that there was no significant difference; ^∗∗^ *p* < 0.01 versus the normal group; ^##^ *p* < 0.01 versus normal group + challenge HDM; ^Δ^ *p* < 0.05; ^ΔΔ^ *p* < 0.01 versus allergic rhinitis group. Each bar shows the mean ± SEM of 4 mice/group. Statistical tests were performed using a one‐way ANOVA. HDM, house dust mite allergen extract; IT, HDM immunotherapy.

**Figure:**
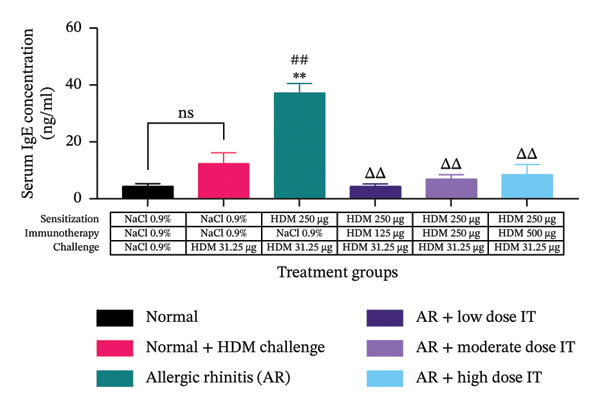
(a)

**Figure:**
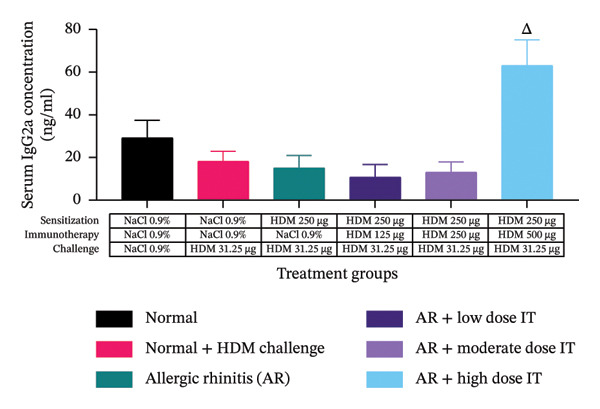
(b)

**Figure:**
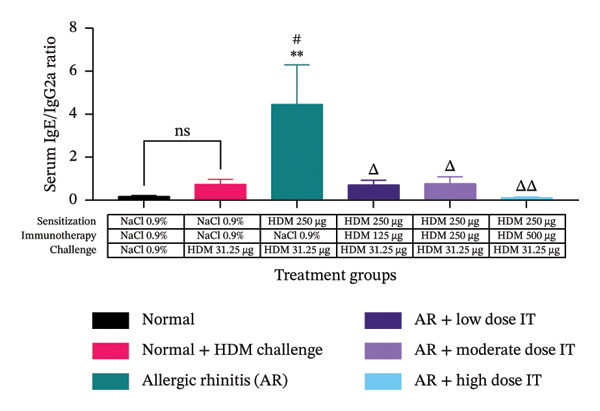
(c)

After obtaining the values for IgE and IgG2a serum levels in the mice, the ratio of IgE/IgG2a was calculated for each treatment group. The ratios of serum IgE/IgG2a in the group of AR mice administered HDM‐IT at low (0.712 ± 0.211), moderate (0.779 ± 0.311), and high (0.112 ± 0.035) doses showed a lower serum IgE/IgG2a ratio compared with the group of untreated AR mice (4.466 ± 1.830; *p* < 0.05, *p* < 0.05, and *p* < 0.01, respectively) (Figure 5(c)).

## 4. Discussion

AR is a worldwide health challenge that demands effective and sustainable treatment strategies. At present, AIT is the only causative approach capable of modifying the course of disease. This study explores the desensitization mechanism of HDM‐based AIT in a mouse model of AR. Establishing a relevant and reliable in vivo model is essential for exploring disease pathogenesis and therapeutic effects before translation to humans. While desensitization mechanisms of AIT have been studied in mouse models of allergic asthma [33, 34], fewer studies have addressed subcutaneous HDM‐IT in AR.

This study successfully established a robust mouse model of AR induced by HDM, as evidenced by the consistent manifestation of clinical symptoms, including increased nose rubbing and sneezing, together with immunological hallmarks such as elevated IL‐4 mRNA expression, enhanced eosinophil infiltration in the nasal mucosa, and increased serum IgE levels. These findings validate our sensitization and challenge protocol as a reliable platform for evaluating the efficacy and mechanisms of AIT.

The inclusion of the Normal + HDM Challenge group provided an important control for nonspecific nasal responses. A direct comparison between the Normal + HDM Challenge group and the AR group further emphasizes the critical role of systemic sensitization in disease development. Although both groups were exposed intranasally to HDM, only the AR mice developed a chronic Th2‐biased immune response. In contrast, the Normal + HDM Challenge mice did not exhibit allergic reactions or persistent immunopathology. This distinction emphasizes that AR pathophysiology arises not merely from allergen exposure but from aberrant adaptive sensitization.

Furthermore, administering HDM‐IT in AR mice at all doses decreased the nose‐rubbing and sneezing response compared with untreated AR mice. These results align with prior reports showing that *Phleum pratense* (timothy grass) extracts administered sublingually reduced sneezing in AR mice [35] and with clinical findings that HDM‐IT improves long‐term AR symptoms in humans [36, 37].

**FIGURE 6:**
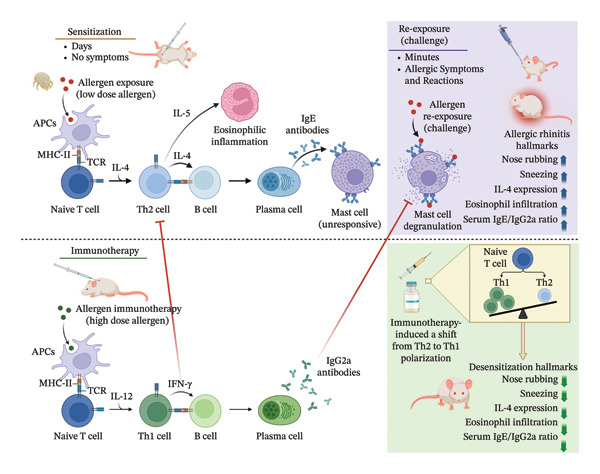
Proposed desensitization mechanisms of allergen (HDM)‐IT in treating AR. The mechanism of desensitization revealed in our study is fundamentally anchored in immune deviation. The robust upregulation of HDM‐specific IgG2a, alongside the suppression of IL‐4, confirms a systemic repolarization of a proallergic Th2 phenotype toward a Th1‐dominant response. Murine IgG2a is functionally equivalent to human IgG1, both being Th1‐associated isotypes capable of high‐affinity antigen binding. In the context of immunotherapy, the induction of these high‐titer IgG antibodies confers protection primarily through competitive antagonism. By outcompeting low‐affinity IgE for allergen binding, IgG2a effectively masks’ the allergen, preventing the cross‐linking of FcεRI on effector cells (mast cells). This steric blockade inhibits the immediate hypersensitivity cascade, resulting in the sustained attenuation of nasal symptoms observed in the high‐dose treatment group. TCR: T cell receptor; MHC: Major Histocompatibility Complex; APCs: antigen‐presenting cells and FcεRI: high‐affinity IgE receptor.

Consistent with a decrease in nose‐rubbing and sneezing behavior, we also found that the administration of HDM‐IT to AR mice decreased IL‐4 mRNA expression in the nasal mucosal tissue. Several previous studies, both in animals and humans, revealed that the success of IT in inducing tolerance/desensitization to allergens was associated with a shift in the immune response from Th2 inflammation towards Th1 inflammation and suppression of IL‐4, which correlated with an improvement in clinical allergy symptoms (Figure 6) [35, 38, 39].

We also observed that another proinflammatory biomarker, eosinophil infiltration, declined following HDM‐IT. Moderate‐ and high‐dose groups of AIT showed reduced eosinophil counts compared with untreated AR mice, while the low‐dose group showed no significant reduction. These results parallel recent findings in which epicutaneous HDM‐IT via microneedles lowered nasal eosinophil infiltration in AR mice [40]. Reduced eosinophilia by AIT likely reflects both suppression of Th2 cells and induction of Th1 and Th2 activities, as both pathways limit IL‐5‐dependent eosinophil maturation, proliferation, activation, and migration [15, 20, 41–43].

In addition, the administration of HDM‐IT in AR mice at all doses resulted in significantly lower serum IgE levels than control AR mice. These results indicate that HDM‐IT effectively inhibits the increase of serum IgE levels. These findings are consistent with those of previous studies evaluating AIT in allergy‐tested animals, using allergens such as *Phleum pratense*, rBet v 1 (a recombinant of an allergen derived from birch pollen), and HDM [35, 40, 44, 45]. According to the PRACTALL consensus, sustained administration of AIT consistently reduces serum IgE [46].

IgG2a was chosen as another biomarker that represents the humoral immune response because, in BALB/c mice, it reflects IFN‐γ–driven class switching and is considered a reliable surrogate of Th1 polarization. Enhanced allergen‐specific IgG2a indicates a deviation toward Th1 responses, which can antagonize Th2‐driven inflammation and mitigate IgE‐mediated pathology [47–49]. In this study, the AR mice that received a high dose of HDM‐IT but not low or moderate doses exhibited a significant increase in serum IgG2a levels compared with the untreated control group. These findings confirm that the effect of HDM‐IT at increasing serum IgG2a levels is dose‐dependent. Similar results were found in mice models of allergic asthma that were sensitized intranasally to HDM extract for 5 weeks. They showed that IT with 5 mg HDM extract, but not 0.5 mg (dose‐dependent), induced higher serum IgG2a levels than untreated allergic asthmatic mice [50].

Furthermore, an analysis of the serum IgE/IgG2a ratios in this study revealed that the AR mice administered HDM‐IT at all doses showed a significantly lower serum IgE/IgG2a ratio than untreated mice. These changes may reflect a shift in the immune response balance from Th2 to Th1 (Figure 6) [45, 50].

A shift in the immune response from a proallergenic T‐cell response (generated by Th2) towards a subset that inhibits Th2 (i.e., Th1 and/or Treg) is a hallmark of successful AIT [17, 51]. Th2 cells drive allergy by producing IL‐4, which promotes IgE production and suppresses Th1 responses, and IL‐5, which regulates eosinophil growth and differentiation [52, 53]. In contrast, Th1 cells produce IFN‐γ and IL‐2, which inhibit Th2 activity [17, 54]. And stimulate IgG2a production in mice [55]. This results in a reduced IgE/IgG2a ratio, as observed here and in other reports (Figure 6) [45, 56, 57].

Taken together, this study is in line with previous studies employing HDM‐based AR primarily focused on establishing experimental protocols and demonstrating therapeutic efficacy, as reflected by reductions in nasal symptoms and broad Th2 cytokine suppression [58, 59]. However, these previous studies utilized relatively mild sensitization protocols to optimize screening workflows [58, 59]. Consequently, their immunological assessments were largely descriptive, focusing on symptom improvement and IgG1 responses without addressing the pharmacological requirements for desensitizing severe or refractory phenotypes [58, 59].

In contrast, the present study was designed to dissect the mechanistic basis of immunotherapy‐induced tolerance in a high‐load sensitization model (250 μg HDM). By applying graded doses (125 μg, 250 μg, and 500 μg), we demonstrate that effective desensitization in this hypersensitized state is strictly dose‐dependent. Notably, increasing immunotherapy doses to 500 μg resulted in progressive suppression of nasal eosinophilic inflammation and IL‐4 expression, indicating that a high‐zone therapeutic threshold is required to modulate local Th2‐driven pathology in severe allergic conditions.

At the systemic level, our analysis extends beyond IgG1 by demonstrating a significant induction of HDM‐specific IgG2a and a marked reduction in the IgE/IgG2a ratio. This shift provides quantitative evidence of immune deviation toward a tolerogenic Th1‐associated profile. Importantly, these immunological changes were functionally linked to sustained protection under repeated intranasal challenges. Unlike previous studies that assessed outcomes shortly after treatment, our design confirms that this specific Ig reprogramming confers durability against persistent environmental allergen exposure. Collectively, these findings distinguish our work by moving beyond the establishment model toward a mechanistic understanding of high‐dose desensitization strategies for sustaining immune tolerance.

Although the present study demonstrates that HDM immunotherapy effectively attenuates Th2‐driven inflammation, several mechanistic aspects warrant further investigation. In this study, IL‐4 was selected as the primary cytokine readout because of its upstream and nonredundant role in Th2 polarization and IgE class switching. However, additional Th2‐associated cytokines such as IL‐5 and IL‐13, which play key roles in eosinophil survival, recruitment, and mucus‐associated pathology, were not directly assessed at the transcriptional or protein level in the nasal mucosa. Likewise, Th1‐related cytokines, including IL‐12 and IFN‐γ, were not measured during immunotherapy, limiting direct evaluation of local Th1 immune activation. Despite these limitations, the significant reduction in eosinophil infiltration in the nasal mucosa, together with marked suppression of systemic IgE levels and dose‐dependent induction of IgG2a, supports a functional shift away from a Th2‐dominant immune response. Nevertheless, comprehensive tissue‐level cytokine profiling and cellular phenotyping will be necessary in future studies to fully delineate the molecular and cellular pathways underlying HDM‐IT–induced immune deviation and tolerance.

Finally, while the present study provides robust mechanistic insights into the efficacy of HDM‐IT in modulating localized (nasal mucosa) and systemic (serum) immunoregulatory responses, several avenues warrant further investigation. Future studies should focus on secondary lymphoid organs, particularly the cervical draining lymph nodes and the spleen, to fully elucidate the compartmentalized priming and expansion of allergen‐specific regulatory T cells (Tregs) and regulatory B cells (Bregs). Furthermore, evaluating the ’sustained unresponsiveness’, the persistence of clinical tolerance long after the cessation of immunotherapy, will be crucial for defining the long‐term memory of this desensitization network. Finally, employing advanced high‐resolution methodologies, such as single‐cell RNA sequencing (scRNA‐seq) of the nasal mucosa, could provide an unprecedented map of the local microenvironmental niches and rare resident immune subsets that orchestrate mucosal tolerance. Addressing these aspects will accelerate the clinical translation of HDM‐IT strategies for allergic rhinitis.

## 5. Conclusion

Overall, this study demonstrates that the administration of HDM‐IT is effective at inducing a tolerance/desensitization response to HDM allergens. This was evidenced by reduced nasal symptoms (fewer nose‐rubbing and sneezing episodes), suppression of IL‐4 mRNA expression, diminished eosinophil infiltration in nasal mucosa, lower serum IgE levels, increased serum IgG2a levels, and decreased serum IgE/IgG2a ratios. Together, these findings highlight the potential of HDM‐IT to rebalance Th2‐driven inflammation and promote protective immune responses.

## Funding

This research was funded by Airlangga University with EQUITY‐International Research Network Scheme 2025 No. 1553/UN3/2025.

## Ethics Statement

All experiments were performed at the Laboratory of Animal Research, Faculty of Pharmacy, Universitas Airlangga, in accordance with the Declaration of Helsinki and approved by the Research Ethics Committee of the Faculty of Veterinary Medicine, Universitas Airlangga, Surabaya, Indonesia, with a certificate of ethical feasibility (No. 2.TO058.05.2021).

## Conflicts of Interest

The authors declare no conflicts of interest.

## Data Availability

Data will be made available by the corresponding author upon reasonable request.
